# AGA-PancreasFest Joint Symposium on Exocrine Pancreatic Insufficiency

**DOI:** 10.1016/j.gastha.2022.11.008

**Published:** 2022-11-15

**Authors:** David C. Whitcomb, Sinead N. Duggan, Robert Martindale, Mark Lowe, Virginia A. Stallings, Darwin Conwell, Jodie A. Barkin, Georgios I. Papachristou, Sohail Z. Husain, Christopher E. Forsmark, Vivek Kaul

**Affiliations:** 1 Division of Gastroenterology, Hepatology and Nutrition, Department of Medicine, University of Pittsburgh, Pittsburgh, Pennsylvania; 2 Department of Cell Biology and Molecular Physiology, University of Pittsburgh, Pittsburgh, Pennsylvania; 3 Department of Human Genetics, University of Pittsburgh, Pittsburgh, Pennsylvania; 4 Department of Surgery, School of Medicine, Trinity College Dublin, Tallaght University Hospital, Dublin, Republic of Ireland; 5 Department of Surgery, Oregon Health and Science University, Portland, Oregon; 6 Department of Pediatric Science, Washington University School of Medicine, St. Louis, Missouri; 7 Children’s Hospital of Philadelphia, University of Pennsylvania Perelman School of Medicine, Philadelphia, Pennsylvania; 8 Department of Internal Medicine, University of Kentucky College of Medicine, Lexington, Kentucky; 9 Division of Digestive Health and Liver Diseases, Department of Medicine, University of Miami, Leonard M. Miller School of Medicine, Miami, Florida; 10 Division of Gastroenterology, Department of Medicine, Ohio State University Wexner Medical Center, Columbus, Ohio; 11 Division of Gastroenterology, Hepatology, and Nutrition, Department of Pediatrics, Stanford School of Medicine and Stanford Medicine Children's Health, Stanford, California; 12 Division of Gastroenterology, Hepatology and Nutrition, Department of Medicine, University of Florida, Gainesville, Florida; 13 Division of Gastroenterology & Hepatology, Department of Medicine, University of Rochester Medical Center, Rochester, New York

**Keywords:** Maldigestion, Malnutrition, Pancreatic Enzyme, Chronic Pancreatitis

## Abstract

Exocrine pancreatic insufficiency (EPI) is a clinically defined syndrome based on the physician’s assessment of a patient’s maldigestion. However, current clinical definitions are inadequate in determining (1) the threshold of reduced pancreatic digestive enzyme secretion that determines “pancreatic insufficiency” in an individual patient; (2) the role of pancreatic function tests; (3) effects of differing metabolic needs, nutrition intake, and intestinal function/adaptation (4) when pancreatic enzyme replacement therapy is needed; and (5) how to monitor and titrate multiple therapies. Experts and key opinion leaders were invited to *PancreasFest 2021* to discuss and help clarify mechanistic issues critical to defining EPI and to address misconceptions and barriers limiting advancements in patient care. Clinically EPI is defined as inadequate delivery of pancreatic digestive enzymes to meals to meet nutritional needs and is reversed with appropriate treatment. A new mechanistic definition of EPI was proposed that includes the disorders *essence* and *character*: (1) EPI is a disorder caused by failure of the pancreas to deliver a minimum/threshold level of specific pancreatic digestive enzymes to the intestine in concert with ingested nutrients, followed by enzymatic digestion of a series of individual snacks and meals over time to meet nutritional and metabolic needs, given (a) the specific macronutritional and micronutritional needs; (b) nutrient intake; (c) exocrine pancreatic function; and (d) intestinal anatomy, function, diseases, and adaptative capacity. (2) EPI is characterized by variable deficiencies in micronutrients and macronutrients, especially essential fats and fat-soluble vitamins, by gastrointestinal symptoms of nutrient maldigestion and by improvement or correction of nutritional state with lifestyle changes, disease treatment, optimized diet, dietary supplements, and/or administration of adequate pancreatic enzyme replacement therapy. EPI is complex and individualized and multidisciplinary approaches are needed to optimize therapy. Better pancreas function tests and biomarkers are needed to diagnose EPI and guide treatment.

## Introduction

Patients with pancreatic disease typically develop exocrine pancreatic insufficiency (EPI) as the severity of the disease or extent of resections increase. However, there is tremendous variability among patients in terms of symptoms, nutritional consequences, compensatory mechanisms, and clinical trajectory.^1^ This problem is confounded by poor understanding and/or adherence to published treatment guidelines.^2^ Furthermore, methods for customized holistic nutrition needs assessment and selection are lacking and timing and dose of treatments remain suboptimal. Together, these issues can lead to poor nutrition, low quality of life, and even reduced survival in some cases.

To address these questions, a group of physicians, scientists, dietitians, and other key opinion leaders were invited to present their opinions and provocative ideas, with supporting evidence to challenge current *ad hoc* approaches and provide a vision for the future. A virtual symposium was held on July 23, 2021, co-sponsored by the *American Gastroenterology Association* and *PancreasFest 2021* (PF21) with international, on-line participation (Supplemental information). Many gaps and barriers were identified (Table 1). The highlights of the symposium are summarized here, with a draft proposal for a new mechanistic definition of EPI presented at the end of this document.

**Table 1:** Clinical and Research Gaps Related to EPI

The lack of a widely available, accurate, and feasible test for exocrine pancreatic insufficiency (ie, pancreatic function test). • Clinical features are nonspecific with substantial overlap with SIBO, IBS, celiac disease, and many others. • Quantitative measures of nutrient digestion and absorption (eg, 72-h fecal fat test) are cumbersome and do not prove that fat maldigestion is actually due to pancreatic insufficiency. • Measuring pancreatic enzyme output through tube-based or endoscopic-based pancreatic function tests is generally not feasible for clinical practice. • Breath tests which measure pancreatic enzyme-specific digestion are available in Europe. They have a long test duration (eg, 6 h) while sitting still that is not acceptable to many patients. • Fecal elastase testing has a high false positive rate and is prone to errors if not done on a solid or semi-solid stool specimen. The poor correlating of this test with other measures of pancreatic function also raise questions as to whether elastase is the best surrogate for all other pancreatic digestive enzymes.
There are numerous clinical situations in which exocrine insufficiency may develop, but determining who requires therapy with pancreatic enzyme replacements is challenging. • acute and chronic pancreatitis • cystic fibrosis • after pancreatic surgery • pancreatic cancer • acid destruction of digestive enzymes • reduced hormonal signaling due to duodenal or jejunal diseases • after intestinal surgery • others? Critically ill, diabetic, extremes of age, etc.
There are nutritional and metabolic consequences of exocrine insufficiency, but these are often not identified and the best clinical strategies to prevent them are undefined. • fat soluble vitamins • osteopenia and osteoporosis
New dietary agents to improve nutrition in cystic fibrosis hold promise but the clinical situations in which they are most useful need further study.
Best management strategies are defined in multiple practice guidelines but compliance with these guidelines is suboptimal and individualization of treatment is undefined. • PERT dose • PERT timing • assessment for vitamin and trace element deficiency • assessment for osteoporosis and osteopenia • assessment for sarcopenia • lifestyle modifications
Very limited patient-reported outcome measures are available to track improvement.
Dietary intervention studies and how diet interacts with EPI
Role of physical activity and exercise in diseases associated with EPI
Need for a mechanistic understanding of the basis for EPI in nonpancreatic disorders, including celiac disease and IBD

## Background

The exocrine pancreas plays an essential role in the digestion and assimilation of nutrients. Progressive loss of pancreatic exocrine function may result in various types of malnutrition of both macronutrients (eg, protein, essential fats) and micronutrients (especially fat-soluble vitamins). It may result in distressing signs and symptoms of maldigestion such as bloating, increased intestinal gas, and steatorrhea. Finally, it may affect other short-term and long-term factors that contribute to poor health and lower quality of life, such as altered calcium metabolism resulting in osteopenia and bone fractures, difficulty-to-control diabetes or sarcopenia resulting in frailty and reduced survival from comorbid conditions.

### The EPI Syndrome

EPI is a syndrome that is difficult to precisely define, diagnose, and properly manage in individual patients, especially those with residual pancreatic function or after pancreatic resection. Among the many challenges of defining EPI as a syndrome is the fact that there are multiple signs and symptoms associated with EPI and all are nonspecific. Furthermore, there are multiple diseases and disorders associated with EPI, but the severity of the underlying disease required to cause EPI is highly variable and may be mitigated by various compensatory mechanisms in the stomach and intestine. Thus, there are no precise definitions or universally accepted diagnostic criteria for EPI. Instead, a “clinical diagnosis” is typically used where clinicians use their own list of evidences and experience to presumptively diagnose EPI.^3^

The term EPI implies a reduction in pancreatic function. However, measuring pancreatic function with traditional exocrine pancreatic function tests (PFTs) is complex, invasive, and not standardized. Thus, the degree of EPI is typically estimated in highly subjective and inaccurate ways.

EPI is also a conditional syndrome. It implies that the reduction in pancreatic enzyme output has fallen below a critical threshold such that an intervention with pancreatic enzyme replacement therapy (PERT) is required to restore nutrient digestion and absorption back to an acceptable range. However, the criteria for determining a threshold have not been accurately defined. The purpose of pancreatic enzymes is to digest an ingested meal. The purpose of meals is to provide nutritional needs. So should EPI be based on the digestion of individual meals or on the integrated effect of digesting multiple meals over time? Should it be based on the maldigestion of specific compounds on serum levels of nutrition biomarkers, deficiencies of certain vitamins, or the development of metabolic disorders?

### Relationship Between Pancreatic Function and EPI

It is widely accepted that the pancreas has a large exocrine reserve and that EPI only occurs when residual pancreatic secretion is < 10% of normal.^4^ This assertion can be traced to a frequently cited 1973 study reporting that pancreatic lipase output to less than 10% for fecal fat excretion to rise.^5^ However, an exploration of the study challenges this assertion. The study in question showed that 16 of 17 chronic pancreatitis patients had lipase secretion < 10% of normal. However, they recruited just one patient with ‘mild/moderate’ EPI and therefore did not show evidence that those with mild or moderate impairment in lipase secretion have normal absorption.^6^ A subsequent study published in Germany in 1986^7^ was regarded as corroboration of the 1973 study; however, they showed that 3 patients with severe EPI (< 10% lipase secretion) had normal fat excretion, while several patients with moderate EPI had steatorrhea. A third study,^8^ also almost 40 years old, examined lipase and colipase secretion in children with Shwachman-Diamond syndrome and cystic fibrosis (CF), comparing them to children with normal secretion as well as Shwachman-Diamond syndrome/CF patients with fat excretion < 7% of measured fat intake (subjects ate ad lib). They demonstrated that those with near zero lipase secretion uniformly had gross fat malabsorption. They described ‘borderline’ subjects with severely reduced lipase secretion who had surprisingly variable fat excretion (from normal to severe fat malabsorption) but proposed that lipase and colipase secretion had to be severely impaired before steatorrhea occurred. In general, there have been insufficient studies (and few, if any, recent investigations) researching this important issue and the claim that the pancreas has a large reserve has not been adequately proven.

This issue matters because EPI is frequently untreated or undertreated^9^ and the prevalent belief that the pancreas must be at least 90% compromised to induce steatorrhea is unhelpful, at best. Regardless, a critical reduction in pancreatic enzyme secretion in an individual will result in the malabsorption of nutrients.^10^ An evidence-based, systematic approach to determining EPI is needed.^11^

The threshold for developing EPI is not only defined by pancreatic exocrine secretory capacity but also by the patient’s nutritional needs, dietary preferences, eating patterns, intestinal structure and function, and other comorbidities. Thus, nonexocrine pancreatic factors that can maintain digestion and absorption are also critical to define and manage.

Within the context of the EPI syndrome, clinicians first want to know how to anticipate, detect, evaluate, and diagnose EPI in their patients. Furthermore, they want to know how to prescribe PERT, when it is indicated, and how to properly dose and monitor it. They often wish to understand complementary strategies to improve nutrition through diet and various supplements. Finally, they want to know how to track nutritional status and overall health over time and when adjuvant therapies are needed. This knowledge must be shared and coordinated with other disciplines including dietitians and subspecialists, such as gastroenterologists, endocrinologists, and surgeons.

## Definitions of Exocrine Pancreatic Insufficiency, Malnutrition, Maldigestion, and Malabsorption

### Definition of EPI

EPI has recently been defined as “a reduction in the quantity and/or activity of pancreatic enzymes to a level that is inadequate to maintain normal digestive processes”^12^ or “a reduction of the pancreatic exocrine activity in the intestine at a level that prevents normal digestion.”^13^ These descriptive definitions, like most others, provide little clarity on patient nutritional needs and variables that markedly alter the threshold of “pancreatic insufficiency” based on diet, intestinal integrity, adaptation, and comorbidities. Based on these limitations, a new mechanistic definition of EPI is proposed (section Draft mechanistic exocrine pancreatic insufficiency definition).

Nutrition is also an umbrella term covering assimilation and utilization of all the metabolic and physiologic needs of living organisms under a variety of conditions. Based on this more holistic view, the threshold for EPI is a function of (1) the nutritional needs of the patient, considering growth, comorbidities, and chronic inflammatory/catabolic states; (2) dietary intake, considering food accessibility, culture, specialized diets, nutritional value of selected foods; (3) residual exocrine pancreas function (discussed below); and (4) the absorptive capacity of the intestine as affected by normal or postsurgical anatomy, mucosal function, motility, inflammation, the microbiome, and physiological adaptation. An updated definition of EPI is needed to address both the state of pancreatic function and extrapancreatic factors. Furthermore, the threshold for diagnosing EPI requires a global assessment of the patient.

### Malnutrition, Maldigestion, and Malabsorption Related to EPI

EPI is typically suspected in the context of known pancreatic pathology and specific macronutritional or micronutritional deficits or distressing clinical symptoms of abdominal pain, bloating, flatulence, diarrhea, steatorrhea, or unintentional weight loss. Pancreatic digestive enzymes play an important role in one step of the ingestion-assimilation pathway. EPI contributes to malnutrition because of maldigestion, with signs and symptoms aggravated by malabsorption.

*Malnutrition* in pancreatic disease, such as chronic pancreatitis, typically consists of a combination of reduced food intake, impaired digestion, and assimilation that is confounded by acute and chronic inflammation leading to both altered body composition and diminished biological function.^14^

*Maldigestion* represents insufficient or incomplete breakdown of nutrients in the gastrointestinal tract, often due to a lack of specific digestive enzymes at different locations within the gastrointestinal tract. Lack of pancreatic digestive enzymes is compensated by enzymes from the mouth to the colon that digest carbohydrates, proteins, and some fats, with the exception of the digestion of specific fats that require pancreatic digestive enzymes (section Nutrient digestion by pancreatic enzymes). Diseases of the mouth (eg, poor dentition), stomach, intestine (lactose intolerance, sucrase-isomaltase deficiency), and various surgeries may also contribute to maldigestion (sections Extra-pancreatic variables affecting digestion, absorption, and nutrition and Other disorders with EPI-like features).

*Malabsorption* describes an insufficient or impaired transport of nutrients from the intestinal lumen into the body. Some nutrients are absorbed in specific locations so that regional intestinal diseases such as celiac disease, Crohn’s disease, various enteropathies, or surgical removal or bypass will cause selective or specific deficiencies. Genetic variants can contribute to maldigestion and malabsorption (section Carbohydrate digestion at the brush border). Dysmotility, toxins, small intestine bacterial overgrowth, and other disorders may also affect nutrient absorption.

The complete assessment of EPI is not just to diagnose the pancreatic exocrine deficiency component but to carefully consider all factors and conditions in the gastrointestinal tract that may contribute to nutritional deficits in a patient. These considerations must be factored into treatment and monitoring plans.

## Nutrient Digestion by Pancreatic Enzymes

The pancreas expresses and secretes a wide variety of digestive enzymes targeting multiple components of proteins, carbohydrates, fats, phospholipids, and nucleic acids.^15^ Other parts of the digestive system also express digestive enzymes that can, to a large degree, compensate for loss of pancreatic secretory function. The exception is fat digestion, where the pancreas plays an essential role. Clinically relevant issues in fat digestion and absorption are especially important in CF and much of the landmark clinical studies have been done in these patients.

### Mechanisms of Fat Digestion and Absorption

Dietary fats are essential for good health.^16^ They provide an important source of calories, contribute building blocks for multiple hormones, and improve absorption of fat-soluble vitamins. Triglycerides comprise about 95% of dietary fats. The assimilation of triglycerides requires hydrolysis by lipases to produce fatty acids and the uptake of digestion products by intestinal enterocytes, which require bile salts and an intact intestine.^17^ The digestion of triglycerides begins in the stomach where gastric lipase cleaves 15%–20% of acyl chains. Digestion continues in the duodenum where pancreatic lipases take hydrolysis to completion resulting in the absorption of 95% of dietary fat with 5% or less excreted in the stool.^16^ The predominant pancreatic lipase is colipase-dependent pancreatic triglyceride lipase. The pancreas secretes other lipases including carboxyl ester lipase (CEL), pancreatic triglyceride lipase related protein 2, and phospholipase A2. The roles of CEL and pancreatic triglyceride lipase related protein 2 remain undefined in humans. Phospholipase A2 contributes to the digestion of phospholipids. Deficient secretion of lipases in chronic pancreatitis leads to maldigestion of dietary fats and therefore, steatorrhea, a hallmark of EPI.

### Special Nutritional Needs in Patients With Cystic Fibrosis

Nutrition is a major challenge in children and adults with CF.^18^ Severe EPI occurs in about 85% of patients with CF (CF Foundation Patient Registry 2020 Annual Data Report) and requires life-long PERT. PERT supports digestion and absorption of nutrients, especially dietary fats needed for calories, essential and nonessential fatty acids, and fat-soluble vitamins. PERT improves but does not normalize fat absorption, GI symptoms, and quality of life in most CF patients.

### Treatments That May Reverse EPI

The treatment of celiac disease is a prime example of improvement in EPI with treatment of a confounding disorder (section Diseases with features overlapping with EPI). In patients with pancreatitis from CF and some remaining pancreatic function, treatment of some genetic variants in the cystic fibrosis transmembrane conductance regulator (CFTR) with CFTR modulators resulted in highly significantly improved lung function and weight gain in almost all patients.^19^ In some participants with milder EPI, there was also an unexpected improvement of pancreatic function documented by increased fecal elastase-1 concentrations suggesting that some of the nutritional improvement was from restored pancreatic function.^20^, ^21^, ^22^, ^23^

### New Therapies to Enhance Fat Absorption

Some patients with CF fail to digest and absorb sufficient fats despite high-dose PERT. A new product, an oral oral structure lipid medical food (Encala, Envara Health) was recently developed and tested in a National Institute of Health–funded clinical trial in children and young adults with CF and EPI. Encala is based upon a highly absorbable structured lipid technology with lysophosphatidylcholine and fatty acids and does not require lipase or bile acids for digestion and absorption. In the randomized, active placebo, double-blind trial, the participants in the Encala treatment group had significantly improved fat absorption test and the clinically important outcomes of weight, height, and essential fatty acid status at 3 and 12 months.^24^, ^25^, ^26^ Encala is used for nutrition support in patients with pancreatic, liver, and intestinal diseases and the general experience from care teams and patients support the trial data with clinically meaningful weight gain and reduced gastrointestinal symptoms in both children and adults as shown in published data^24^, ^25^, ^26^ and documented in a company early experience patient survey and reported informally by registered dietitians using the product in clinical care.

## Extra-pancreatic Variables Affecting Digestion, Absorption, and Nutrition

The majority of patients with advanced pancreatic disease have maldigestion and malnutrition and most of these patients can be managed successfully. The key elements of management are PERT to treat decreased enzyme secretion, optimal glycemic control timed with meals and PERT, and vitamin and trace element supplementation. In the future, there may also be a role for probiotics or otherwise managing the microbiome.^27^

There are multiple challenges to address regarding short-term and long-term nutrition in pancreatic disease and these challenges are confounded by the fact that there are no adequate nutritional markers: “if you cannot measure it, you cannot improve it.”^28^^,^^29^ One common measure is body mass index (BMI); however, morbidly obese people can also be malnourished (sarcopenia).

Nutrition in patients with pancreatic disease is dependent on nutritional needs, diet, pancreatic exocrine and endocrine function (ie, capacity to secrete pancreatic digestive enzymes and pancreatic hormones in response to physiological stimuli), and gastrointestinal integrity, anatomy, and gastrointestinal function.^30^ Deficiencies in any of these variables can often be compensated for by physiological reserves or adaptation of the other variables, up to a point. In this section, we will focus on gastrointestinal tract deficiencies and adaptation in the context of pancreatic disease and reduced pancreatic digestive enzyme production.^30^

### Enzymes of the Intestinal Mucosa

A key aspect of the gastrointestinal tract is the intestinal mucosal brush border, including epithelial cells with microvilli that provide a massive surface area (about 32 m^2^)^31^ and redundancy for luminal digestive enzyme absorption and function. This digestive and absorptive reserve capacity is significant because only about 100 cm of intestines is needed before the patient develops short gut syndrome, requiring life-long parenteral nutrition (PN). While intestinal length is important, the region of the gut that is lost is also important as different parts may serve specific functions.^32^

### Carbohydrate Digestion at the Brush Border

The gut digests and absorbs nutrients at the brush border, whereas pancreatic digestive enzymes act within the milieu of the intestinal lumen. Clinically important examples of specific enzymes that may be deficient through genetic or genomic mechanisms include lactase (digests lactose to galactose and glucose) and sucrase-isomaltase (SI). The SI complex is essential for the digestion of dietary carbohydrates including starch, sucrose, and isomaltose, and genetic deficiencies of this enzyme causes carbohydrate maldigestion.^33^^,^^34^ Most dietary starches are readily hydrolyzed to simple sugars and absorbed through active transport. Under normal conditions, the carbohydrate absorption capacity of the small intestine is massive and the brush border enzymes can compensate for lack of pancreatic amylase.^35^ In summary, EPI is not associated with carbohydrate malnutrition.

### Protein Digestion at the Brush Border

Protein digestion is complex and typically uses the stomach, pancreas, and small intestine in different ways. The stomach plays a key role in denaturing proteins with hydrochloric acid and initiates digestion of animal proteins with pepsin, essentially unraveling the tertiary structure of proteins to allow pancreatic enzymes target sites for cleavage of the peptide chain. Pancreatic digestive enzymes are primarily proteases with both endopeptidases (eg, the trypsins, chymotrypsins, elastases) and exopeptidases (eg, carboxypeptidase A, B, etc.).^15^ The small intestine mucosa expresses aminopeptidases, dipeptidyl aminopeptidases, and dipeptidases and absorbs both single amino acids and small peptides up to 3 amino acids in length through multiple mechanisms. About 70% of protein mass following a meal is absorbed as dipeptides and tripeptides via PepT1 (SLC15A1), an intestinal hydrogen peptide cotransporter.

The body adapts to the protein absorption and utilization based on metabolic needs (anabolism), pre-existing and current disease (catabolism), age, sex, quality of protein, route of protein delivery, percent splanchnic extraction, status of intestinal microbiome, and even dietary patterns.^36^^,^^37^ Anabolic resistance, defined as the failure of normal anabolic stimuli to induce total body net protein synthesis, is common in inflammatory conditions such as pancreatic cancer, cystic fibrosis, and acute and even chronic pancreatitis.^38^^,^^39^ All of these pancreatic conditions commonly demonstrate both EPI and anabolic resistance.^40^ Anabolic resistance leading to sarcopenia has been shown to have a prevalence of 17%–64% in chronic pancreatitis leading to a decrease in quality of life, increased hospitalizations, and even mortality.^41^ In summary, EPI can be associated with protein malnutrition. Dietary strategies may compensate for loss of pancreatic proteolytic digestive enzymes to a significant degree. PERT may be needed to assist in protein digestion based on dietary preferences, intestinal integrity, and comorbidities.

### Surgical Resections and Bypasses of the Stomach and Regions of the Intestine

Major gastrointestinal surgery yields significant alterations in the patient’s ability to digest and absorb lipids and other macronutrients and micronutrients. Fat absorption and digestion following pancreatic surgery depends on several factors including extent of gastric resection, duodenectomy, and proximal jejunal resection or bypass. Bariatric surgery typically results in EPI.^42^ The impact of a pancreaticoduodenectomy (PD) will depend on multiple factors including specifics of the procedure, pylorus sparing versus standard Whipple, and the condition of the pancreas prior to surgery. Exocrine function generally deteriorates following PD. A recent prospective cohort study reported that 20.5% of patients had EPI preoperatively, 51.3 % showed deterioration of exocrine function postoperatively, and that 64.1 % of patients required PERT postoperatively.^43^

Micronutrient deficiency in patients with pancreatic diseases and postpancreatic surgery is multifactorial, resulting from loss of functional pancreatic tissue, inadequate oral intake, loss of the primary absorptive site following a variety of surgical anatomic changes such as Roux en Y and PD, and changes noted in mucosal absorption dynamics from the changes in pH and nutrient mix. Fats are normally absorbed in the jejunum through complex molecular mechanisms,^17^^,^^44^ so surgeries that exclude or bypass the duodenum and jejunum may result in selective deficiencies. Fat soluble vitamin deficiency is more common than water-soluble vitamins. Vitamin B12 absorption is the exception because B12 requires release from R-protein and subsequent intrinsic factor (IF) binding in the duodenum. The B12/IF complex is then absorbed in the distal ileum. In severe EPI following pancreatic resection, B12 binding to IF is disrupted.^45^^,^^46^ Fat soluble vitamins require micelle formation like other fats and with EPI-altered micelle formation is common. Vitamin A deficiency has been reported at 35% and vitamin E at 18%, in most studies with EPI, but they are easily replaced. Vitamin D deficiency, on the other hand, is reported in EPI patients at approximately 70%. In general, routine/ blanket nutrient supplementation is not recommended (rather, vitamins should be measured and deficiency treated accordingly) because hypervitaminosis is known to occur in patients with chronic pancreatitis.^47^ Overt, clinical vitamin deficiency appears to be rare (as opposed to findings of biochemical deficiency), takes years to develop, and tends to present when there is an additional comorbidity, such as chronic alcoholism, celiac disease, diabetes, or postsurgery.^48^ Expert recommendations include screening for vitamin and mineral deficiencies (especially A, D, E, K, B12, folate, magnesium, zinc, and iron studies) at the time of diagnosis of pancreatic disease and annually based on the clinical condition of the patient.^13^^,^^49^

## Differential Diagnosis of EPI

### Diseases With Features Overlapping With EPI

Due to challenges in the diagnosis of EPI and its common chronic symptoms such as abdominal pain, bloating, and diarrhea, there are a number of conditions that may either mimic EPI or be present concomitantly with EPI and/or may affect a response to PERT.^50^^,^^51^ Common conditions which may mimic or have an overlap with EPI include celiac disease, small intestinal bacterial overgrowth, disaccharidase deficiencies, inflammatory bowel disease, bile acid diarrhea, giardiasis, diabetes mellitus (especially longstanding type 1 DM), functional conditions like irritable bowel syndrome (IBS), and others.^12^

These clinical entities should be considered at the time of making a diagnosis of EPI, at the time of dose adjustment of PERT, and at the time of potentially determining a treatment failure of PERT. In these situations, a provider should ask whether a condition mimicking EPI is present either as an alternative or comorbid diagnosis. Before deeming PERT a treatment failure, it is important to ensure adequate dosing and compliance with PERT as these are common challenges.^52^^,^^53^

### Celiac Disease

Celiac disease may both mimic EPI and be a cause of EPI. A recent meta-analysis of 460 patients with newly diagnosed celiac disease noted a pooled prevalence of EPI at diagnosis of 26.2%.^54^ When patients were treated with a gluten-free diet (GFD), the pooled prevalence of EPI dropped to 8% overall, with contrasts between those who remained symptomatic on GFD compared to asymptomatic on GFD (EPI prevalence symptomatic 28.4% vs asymptomatic 3%, *P* < .001). Finally, those with newly diagnosed celiac disease were significantly more likely to have EPI compared to those treated with GFD (*P* = .031). In a separate natural history study of celiac disease, 30% of patients with persistent diarrheal symptoms had EPI treated with PERT and with treatment of celiac disease over time may have had improvement in EPI with an increase in fecal elastase levels.^55^

### Small Intestinal Bacterial Overgrowth

Small intestinal bacterial overgrowth (SIBO) may present with bloating, diarrhea, abdominal pain/discomfort, and steatorrhea in severe cases, similar to EPI.^56^ Small studies of patients with chronic pancreatitis and EPI have noted an approximately 15% prevalence of SIBO in this population.^57^ In a subsequent meta-analysis of 13 studies (518 patients with chronic pancreatitis who underwent SIBO testing), a pooled SIBO prevalence of 38.6% was found, with increased likelihood of SIBO if EPI was also present (OR 2.5), again reinforcing SIBO as both a cause and consequence of maldigestion.^58^

### Other Disorders With EPI-like Features

There are several less common conditions that may be entertained as possible EPI mimickers. Disaccharidase deficiencies of lactase, sucrase-isomaltase (palatinase), and maltase-glucoamylase may present with flatulence, bloating, and diarrhea, with 46.7% of adults presenting with gastrointestinal symptoms having at least 1 disaccharidase deficiency.^59^ EPI should be considered in those with inflammatory bowel disease, with an estimated prevalence of 14% in Crohn’s disease and 22% in ulcerative colitis, albeit limited in generalizability due to the use of fecal elastase as the method of EPI diagnosis.^60^ With that said, older studies using secretin and cerulein (a CCK receptor agonist) in a direct pancreatic function test for EPI diagnosis (below) showed reduced lipase in 58% of Crohn’s disease and 80% of ulcerative colitis patients with 34% of IBD patients having pancreatic duct abnormalities on ERCP.^21^^,^^61^ Finally, evaluation for infectious etiologies should be entertained if there are risk factors, with specific focus on giardiasis, as it may mimic both IBS and EPI symptoms.^62^

**Figure 1:**
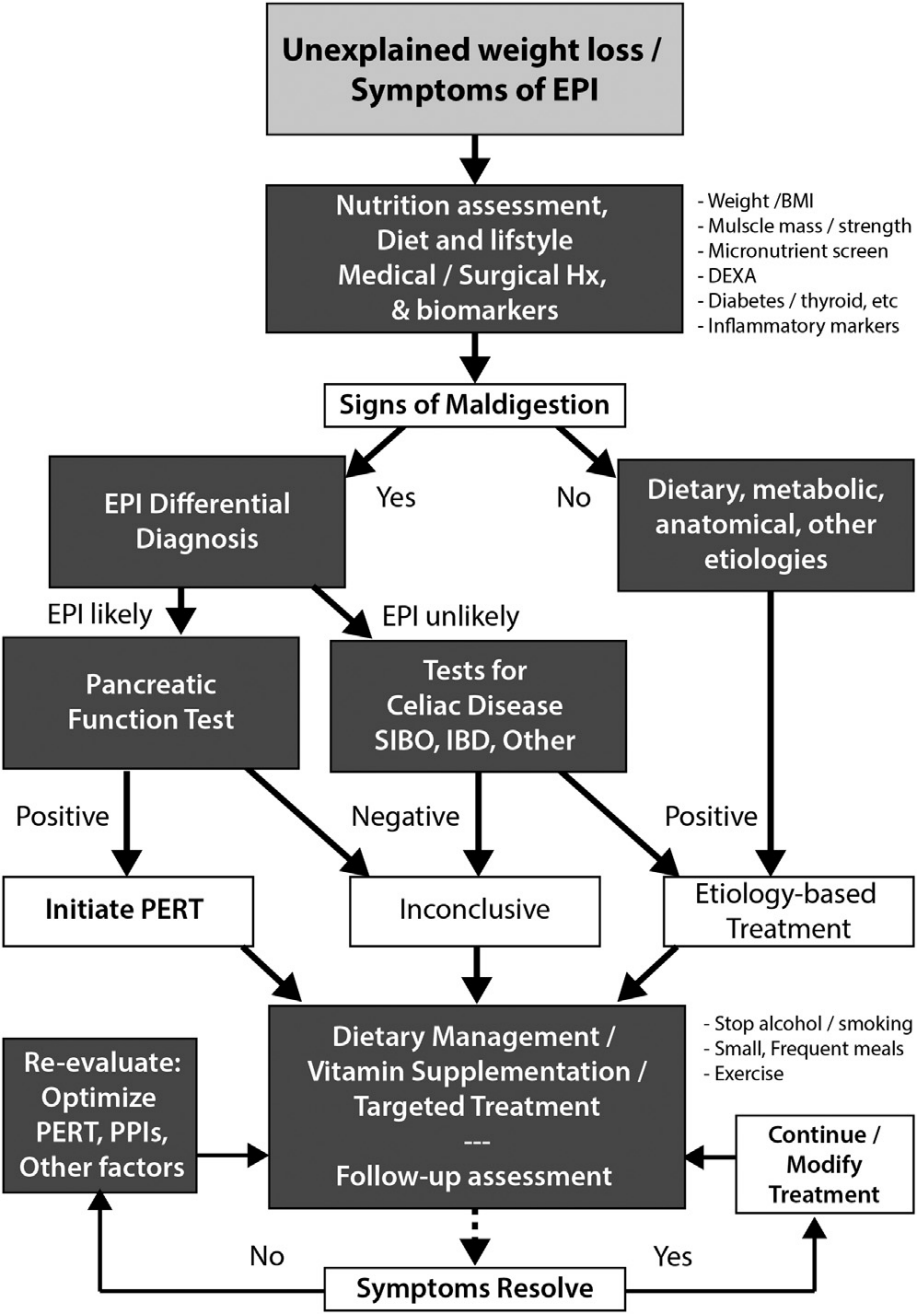
Evaluation and management of suspected EPI. Dark boxes represent evaluation and decision stages leading to rational management of EPI and maldigestion resulting in malnutrition.

In summary, there are several conditions that may produce symptoms similar to EPI and consideration of potential EPI mimickers or comorbid conditions is key to address the underlying pathology and optimize patient therapies. A summary of the approach is given in Figure 1.

**Figure 2:**
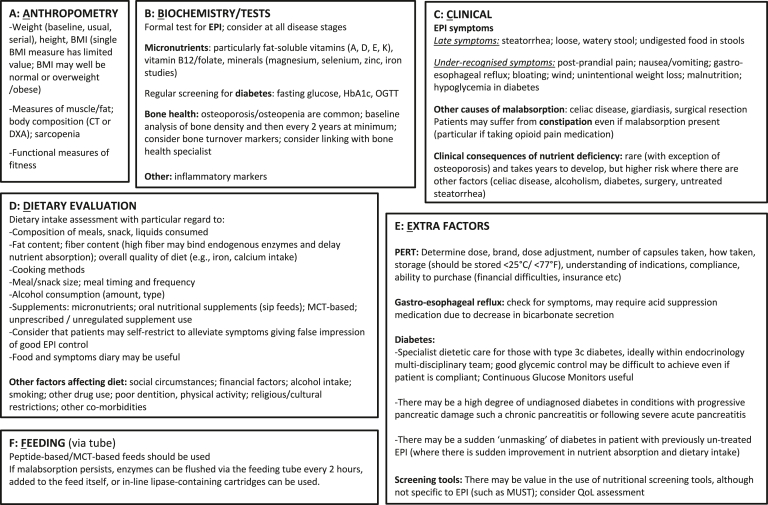
(A–F) Individualized nutrition-related assessment of patients with EPI: Patients should be assessed and regularly monitored by a specialist, experienced pancreatic RD, as part of multidisciplinary management. May also require input from diabetes-specialist RD. CT, computed tomography; DXA, dual x-ray absorptiometry; EPI, exocrine pancreatic insufficiency; HbA1c, glycated hemoglobin; MCT, medium-chain triglycerides; MUST, malnutrition universal screening tool; OGTT, oral glucose tolerance test; PERT, pancreatic enzyme replacement therapy; QoL, quality of life; RD, registered dietitian.

## Pancreatic Function Tests for EPI

There is consensus that exocrine pancreatic function testing (PFT) is important for the diagnosis and management of EPI but that (1) currently, no optimal test exists^63^ and (2) pancreatic function is only one component of digestion and absorption that must be considered in treating or preventing micronutrient or macronutrient malnutrition.

Assessment of exocrine pancreatic function is needed to make an objective diagnosis of EPI in the setting of CP, weight loss after acute or recurrent acute pancreatitis, pancreatic cancer care, after pancreatic resection, in cystic fibrosis, and other rare disorders. PFTs may also be useful in managing other conditions such as long-standing diabetes mellitus (especially with pancreatic atrophy), inflammatory bowel disease, Zollinger-Ellison syndrome, or gastric resection.

The choice of which PFT should be used is complicated and typically differs from center to center. Two categories of test are used: Direct and Indirect PFTs.

### Direct PFT

The most accurate and informative method is the Direct PFT, which uses an oral-duodenal tube (eg, Dreiling tube) or endoscope to collect pancreatic fluid from near the pancreatic duct orifice after stimulation with secretin and/or a CCK analogue to measure bicarbonate (a biomarker of duct/CFTR function) and digestive enzymes (a biomarker of acinar function). Most experts use the endoscopic method.^64^, ^65^, ^66^, ^67^ Bicarbonate levels are the most common biomarker measured, noting that chloride can also be measured as the anionic inverse of bicarbonate. The major limitation of bicarbonate measures is that changes in the level or function of CFTR will alter bicarbonate levels^68^^,^^69^ even if the pancreas is normal and bicarbonate measurement does not always correlate well with other measures of EPI.

A short endoscopic test with a more comprehensive pancreas fluid analysis has been recently described in pediatric patients which measures pancreatic digestive enzyme levels, including amylase, lipase, trypsin, chymotrypsin, and elastase.^70^ Interestingly, loss of enzyme function does not appear to occur in a uniform or linear fashion. In fact, different enzyme activity levels may be lost at different time periods during the natural history of chronic pancreatitis (Conwell, unpublished observations presented at the conference). The greatest limitation of the direct pancreatic function test is that it is invasive (requiring intestinal intubation), requires special training, can be of long duration, and therefore expensive.

### Indirect PFT

Indirect PFTs impute pancreatic function based on biomarkers that are accessible without duodenal intubations. Each common test has benefits and limitations and they often do not correlate with each other.^71^ Imaging test does not correlate with EPI except with advanced pancreatic calcifications and atrophy.^3^ The secretin-stimulated magnetic resonance imaging cholangiopancreatogram (sMRCP) can be valuable in diagnosing CP but the value in diagnosing EPI remains experimental.^72^, ^73^, ^74^, ^75^ In the past, the “gold standard” was fecal fat excretion during a five-day 100-gram fat diet during which the last 3 days on the diet, stool was collected to calculate a coefficient of fat absorption (CFA).^76^, ^77^, ^78^ This test is a burden for patients, expensive, cumbersome, insensitive, and has (or should be) abandoned, although gross steatorrhea still remains an important sign of advanced EPI. The CFA remains the gold standard for pharmaceutical pancreas enzyme replacement trials.

The most widely used test is the fecal human elastase-1 test (FE-1),^1^^,^^4^ although the ScheBo Pancreatic Elastase 1 Stool Test (ScheBo Biotech AG, Giessen, Germany) actually measures chymotrypsin-like elastases (CELA) 3A and 3B (CELA3A and CELA3B).^79^ The test can distinguish normal, mild, and severe EPI under controlled conditions. The utility is limited by the requirement of testing FE-1 in formed stool and poor patient compliance as they often do not collect their stool and return a sample to a testing site. In addition, the test results are not always repeatable (our experience) and there are high false positive rates due to the low prevalence of EPI.

Serum trypsin(ogen) levels are attractive because measuring serum levels of pancreatic digestive enzymes is relatively inexpensive, specific to the pancreas, quantitative, and can be trended over time under appropriate circumstances (eg, flare of acute pancreatitis).^3^^,^^80^ EPI is associated with abnormally low levels. The limitation includes variable “normal” ranges between methods and laboratories it has fallen out of favor.

The ^13^C-mixed triglyceride (^13^C-MTG) breath test appears to be an accurate PFT for triglyceride maldigestion and is widely used in Europe.^4^^,^^42^ Limitations include availability of the ^13^C-substrate, validated detection devises, and the requirement for the subject to sit still in a chair for 6 hours during sample collection.^81^

A new indirect PFT that measure intestinal lipase activity is the Malabsorption Blood Test.^82^ A combination of pentadecanoic acid (PA), a free fatty acid, and triheptadecanoic acid (THA), a triglyceride that requires pancreatic lipase for absorption of the heptadecanoic acid (HA) is ingested and blood samples taken over 9 hours. This test appears to be more sensitive to fat maldigestion than CFA.^83^ Further studies in CF and CP are underway.

The medical community still awaits a clinically useful pancreas function test that is easy to perform, well tolerated by patients, and allows personalized dosing of PERT.

## Nutritional Management of Chronic Pancreatitis

Once the diagnosis of EPI has been confirmed, a general assessment of symptoms, nutritional status, medications, diet, and lifestyle should be performed. This information can then be applied to a multifaceted treatment approach with focus on nutrition, lifestyle modifications, and PERT (Figure 2). Regular follow-up to monitor compliance and response to the treatment plan is necessary to achieve optimal results.

One of the most important components in the assessment of patients with pancreatic disease and potential EPI is a nutritional needs and dietary assessment by a registered dietitian (RD) who has expertise in pancreatic disease and works as a key member of a multidisciplinary pancreas team. RDs have the necessary expertise to assess basal metabolic needs based on patient age, sex, anthropometric measures, and body composition. The metabolic needs are also dependent on growth trajectory (in children), physical activity levels, degree of active inflammation and catabolic state, pathologic weight loss or deficits, body composition, and sarcopenia. Biomarkers of nutritional status are also important (discussed below). RDs should assess dietary intake (accounting for meal/snack size, meal pattern, and frequency), macronutrient (fat, protein, carbohydrates, fiber) and micronutrient (fat-soluble vitamins, calcium, and iron), cooking methods, and the use of oral nutritional and micronutrient supplementation. Dietary intake in chronic pancreatitis is known to be particularly poor, especially among those with alcohol-associated disease.^84^ In rare cases, alcohol-derived calories may make up a large proportion of energy intake. The RD works with the clinicians for management of confounders such as excessive alcohol consumption and smoking, metabolic issues such as diabetes, thyroid disease, cancer, and other medical-surgical comorbidities. This baseline assessment is essential because strategies to modify diet or delivery of nutrients should be considered prior to implementing PERT. Furthermore, nutritional deficiencies should be identified and treated before overt signs and consequences of malnutrition become clinical problems. The components of an individualized nutrition-related assessment for EPI are summarized in Figure 2.

Three important and often overlooked disorders deserve special attention in the context of nutritional management in EPI:

### Inflammaging

Inflammaging (or inflamm-ageing) is a chronic low-grade inflammatory condition initially studied in the elderly, characterized by elevated levels of blood inflammatory markers that appear to carry high susceptibility to chronic morbidity, disability, frailty, and premature death.^85^ This condition may also be important in chronic pancreatitis.^86^^,^^87^ Inflammaging is thought to be driven by chronic systematic inflammation characterized by elevated proinflammatory cytokines (specifically IL6, IL4, TNF, T-cell factor-β, IL8) and low anti-inflammatory cytokines (including IL10).^86^ Systemic inflammation in chronic pancreatitis can be exacerbated by a chronically suboptimal diet, heavy smoking, and EPI; results in an “aged” phenotype; and contributes to osteoporosis and sarcopenia.^88^ Systematic inflammation is also known to be associated with diminished quality of life in patients with chronic pancreatitis using standardised tools (eg, EORTC QLQ-C30. SF-12 and PAN-28) in both general and specific domains including pain, physical functioning, and cognitive functioning.^89^, ^90^, ^91^, ^92^ Clear criteria for diagnosing and measuring the severity of inflammaging are needed so that prospective treatment trials can be designed and tested.

### Osteoporosis

A meta-analysis of 10 studies revealed that two-third of patients with chronic pancreatitis had reduced bone mineral density (osteoporosis or osteopenia) and half of the studies reported a link between low fecal elastase-1 levels and low bone density.^93^ More recently, the requirement for a baseline bone density assessment (dual x-ray absorptiometry, DXA) is included in various guidelines, with a repeat DXA recommended every 2 years.^4^^,^^13^^,^^94^ Patients with chronic pancreatitis should follow basic preventative measures to preserve bone density (physical activity, alcohol/smoking cessation, adequate vitamin D/calcium intake).^13^ However, the optimal pharmacological and lifestyle protocol for treating osteoporosis in chronic pancreatitis is not known and there have been no intervention studies to inform practice.

### Sarcopenia

Sarcopenia refers to the loss of muscle mass, strength, and function.^95^ Sarcopenia occurs more frequently in chronic pancreatitis patients with EPI than without, including in some obese patients where excess fat and low muscle mass co-exist.^96^ In one study of 132 patients with pancreatic disease, sarcopenia was the only clinical factor that was independently associated with EPI.^97^ Prescriptive physical activity may help prevent both osteoporosis and sarcopenia. Surprisingly, there are no studies evaluating the efficacy of physical activity on sarcopenia in chronic pancreatitis.^98^ An older 12-week yoga-based intervention (vs no intervention) study did demonstrate an improvement in quality of life and stress indicators.^99^

Disease outcome is more closely associated with body composition than merely BMI, considering both excess body fat and decreased muscle mass gives a much more accurate evaluation of nutritional status.^28^ The impact of sarcopenia and altered body composition has been demonstrated in chronic pancreatitis, pancreatic and other neoplasia, lymphomas, liver transplantation and intensive care unit patients who are elderly, septic, on extracorporeal membrane oxygenation (ECMO), and/or who have COVID-19 pneumonia.^41^^,^^100^^,^^101^ Therefore, nutritional status is important but nearly impossible to accurately measure by traditional serum levels of visceral proteins such as albumin.^29^ Rather, assessment of nutritional status should incorporate assessments of body composition. Computed tomography (CT)–based body composition assessment could be measured using CT scans that are often readily available for chronic pancreatitis patients. Importantly, functional measurements of muscle strength and physical performance (such as gait speed and handgrip strength) should be done alongside measures of muscle quantity/quality.^102^ Indeed, the importance of routine screening for sarcopenia and including it as a key outcome, even superseding weight and BMI, was highlighted by multiple discussants in the conference.^47^

## Pancreas Enzyme Replacement Therapy: Strategies and Goals

### PERT Formulations

In addition to dietary and lifestyle modifications, PERT is the mainstay of EPI treatment. It has been shown to improve steatorrhea, reduce weight loss, improve postprandial bloating and pain, and improves nutrition^103^ and is safe.^104^ There are several different formulations of PERT approved by the Food and Drug Administration including Creon, Zenpep, Pancreaze, Pertzye, and Viokase (Table 2). Each formulation is available in multiple different strengths and includes proteases, amylase, and lipase, with the dose designation based on lipase content. All are enteric-coated formulas except for Viokase which is a nonenteric coated tablet. Although lipase inactivation occurs when pH levels are less than 4.5, Viokase must be given in conjunction with acid suppression medication. There are several over-the-counter variations of pancreatic enzymes readily available. Although they may be easier to obtain and cost less, patients should be cautioned that their formulation and dosing are neither standardized nor regulated and their efficacy and safety are not known.

**Table 2:** FDA Approved Formulations of PERT

Brand	Formula	Available lipase strengths (USP)
Creon	Enteric-coated microsperes	3000/6000/12,000/24,000/36,000
Zenpep	Enteric-coated beads	3000/5000/10,000/15,000/20,000/25,000/40,000
Pancreaze	Enteric-coated microtablets	2600/4200/10,500/16,800/21,000/37,000
Pertzye	Enteric-coated microspheres	4000/8000/16,000/24,000
Viokase	Nonenteric-coated tablets	10,444/20,880
Relizorb	In-line lipase cartridge	For infusion of tube feed formulas

### PERT Dosing

Typical starting dose of PERT is based on age and weight and is derived from guidelines for EPI treatment in patients with cystic fibrosis (Table 2).^105^, ^106^, ^107^ Of note, these recommendations do not consider many of the other variables discussed here but rather provide an estimate for the “average” subject with severe EPI. For infants, 2000–4000 units of lipase per 4 ounces of formula or breast milk is a reasonable starting dose. In children aged less than 4 years, at least 1000 units of lipase per kilogram (kg) of body weight should be given per meal and 500 units per kg per snack. For children aged 4 years and more and for adults, the typical starting dose is 500 units of lipase per kg per meal (eg, 40,000 U for an 80-kg patient) and 250 units of lipase per kg (20,000 U for an 80-kg patient) per snack. This dose should be titrated up as needed to reduce steatorrhea or gastrointestinal symptoms of maldigestion with optimization based on a more holistic management plan. The maximal dose is 2500 units of lipase per kg per meal or 10,000 units of lipase per kg per day. For comparison, the normal pancreas secretes approximately 900,000 lipase units per meal (11,250 units/kg for an 80-kg person). Dosing at or below the maximum may be important in patients with CF to reduce the risk of developing fibrosing colonopathy. ^108^

Patients should be educated on the appropriate administration of PERT, including the importance of taking it with food (the prescription should specify to take during the meal or snack). The medication cannot be crushed or chewed as it can cause irritation of the oral mucosa. If the patient is unable to swallow capsules, then capsules containing coated enzyme spheres or microspheres can be opened and the beads sprinkled in a small amount of applesauce. Side effects such as nausea, abdominal cramping, abdominal bloating, diarrhea, and constipation may occur with PERT. Rare adverse events of PERT include fibrosing colonopathy, allergic reactions, and hyperuricosuria.^109^^,^^110^

For patients who are on tube feedings, PERT can be given via tube every 2 hours, added to standard or semi-elemental formula (Phillips, page 8, Box 2).^13^ In addition, there is an in-line cartage containing lipase attached to beads (RELiZORB, Alcresta Therapeutics, Inc.) that digest triglycerides as the formula passes through.^111^

### Barriers to the Use of PERT

There are several challenges associated with PERT. Compliance may be a barrier secondary to frequent dosing, timing with oral intake, and the pill burden. Using higher strength capsules for patients who require larger doses reduces pill burden and may improve compliance, but these larger pills may be more difficult to swallow. Patients may refuse or be unable to take PERT due to religious beliefs (although both Muslims and Jews may have special dispensation to take these life-saving pork products), porcine allergies (although the risk is low^112^), or hyperuricemia (and risk of gout) with very high doses in CF patients.^113^^,^^114^ Cost may also be an issue for some patients and many prescriptions are not filled at all due to cost. Providers should be aware of patient assistance programs available through pharmaceutical companies. These programs help reduce out-of-pocket costs if patients meet specified criteria.

### Monitoring Treatment Response and Compliance

Monitoring of symptoms, such as weight stabilization/gain, improved steatorrhea/diarrhea, and reduced postprandial bloating, pain and flatulence are reliable indicators of effective EPI management. It may be helpful to provide patients with tracking tools to record their compliance to dosing of PERT and frequency of symptoms. Simple measures of muscle strength and function are also useful to track (time up and go test). Objective testing, such as timed fecal fat testing or breath testing while on PERT, are rarely used. It is important to note that pancreatic fecal elastase (eg, FE-1) is not affected by PERT and although it is a reliable diagnostic tool for EPI, it should not be used as a marker of therapeutic response.

For patients with persistent concerns for malabsorption despite dietary and lifestyle modifications and compliance with PERT, there are several options. The dose of PERT should be titrated up as needed (eg, up to 90,000/meal in adults) to achieve appreciable improvement in steatorrhea. Although many of the PERT formulations are enteric coated, addition of a proton pump inhibitor or H2 receptor agonist may help.^115^ If symptoms of EPI persist despite the above, alternative causes of malabsorption should be considered. Evaluation for concomitant celiac disease, SIBO, bile acid diarrhea, giardia infection, malignancy, and IBS-D (based on Rome IV^116^) may be needed (section Nutrient digestion by pancreatic enzymes).

## Management Strategies in Patients With Severely Limited Exocrine Pancreas Function

As opposed to the severe acute pancreatitis, specialized delivery methods (feeding tube or parenteral nutrition [PN]) for metabolic support and nutrient delivery are not commonly needed in the chronic setting for EPI, although deep jejunal feeding may rarely be used for pancreatic rest with food-associated pain. Enteral nutrition via tube feeding is used in less than 5% of chronic EPI patients getting adequate PERT.^117^^,^^118^ This group of patients can usually be fed via feeding tube with a standard formula or (more often) a semi-elemental, peptide-based feeding solution. In severe disease, a formula containing medium chain triglycerides which can be absorbed directly into the portal vein should be considered if fat absorption remains inadequate.

PN is rarely needed for EPI and is only used in about 1% of EPI patients unless other issues affecting intestinal absorption are present. Examples of indications for PN in patients with EPI include pancreatic fistula, gastric outlet obstruction, enterocutaneous fistula, and short bowel syndrome.^119^

### Diet and PERT in Moderately Severe EPI

Patients with mild-moderate chronic pancreatitis will usually be able to follow a ‘normal’ diet. However, as the duration and severity of disease increases, and as EPI worsens, it is likely that they will not tolerate large meal portions and that higher fat food will result in symptoms of malabsorption.

Intestinal adaptation to loss of pancreatic enzymes continues throughout the disease process with considerable variation among patients. Except for fat absorption, brush border enzymes can supplant the requirements for pancreatic enzymes except when there is a severe loss of exocrine function. Manipulation of the diet to improve quality of life is possible and can be achieved by the compliant patient.

Nutritional goals for patients with marginal digestive capacity that can maintain nutrition optimization are:

a. Protein at 1.5 to 2.0 gm/kg/d;
b. Carbohydrate calories should be a relatively high percentage of calories with complex carbohydrate being the majority;
c. Limited highly insoluble fiber intake as it can decrease activity of PERT;
d. Soluble fibers are beneficial in that they produce short-chain fatty acids upon fermentation in the colon which serves to decrease inflammation locally and systemically, help maintain colonic mucosal integrity and immune function, and serving as a caloric source;
e. Dietary fat should not be unnecessarily restricted as very low fat diets become unpalatable making compliance low and these increase the risk of fat soluble vitamin deficiency; and
f. Vitamin and mineral supplementation is recommended at ‘standard’ levels. Doses of vitamins or minerals that greatly exceed the recommended doses are not supported by data and may be harmful.

Patients are advised not to limit diary fat intake unnecessarily and it is crucial that PERT is optimized along with all other strategies (such as acid suppression, ensuring patient understands the need for PERT, current timing of PERT, correct storage of PERT to avoid denaturation of enzymes). Completely ‘fat-free’ diets are neither necessary nor palatable nor are they possible in the long term, rather, PERT (along with adequate acid suppression and other strategies) should be optimized to treat the symptoms. Patients must also be taught that the PERT treats the meal, not the pancreas. So, dosing must be adjusted based on what is eaten (type of food, cooking method, portion size, and additions).

### Diet and PERT in Severe EPI

For those who have difficult-to-treat EPI, other reasons for malabsorptive symptoms should be sought and excluded (section Nutrient digestion by pancreatic enzymes). In the latter stages of disease, as EPI escalates, a lower fat diet may become necessary to alleviate distressing gastrointestinal symptoms. A close working relationship between the treating provider and the RD is crucial so that barriers to optimum nutrient assimilation can be identified, communicated, and overcome. Frequent monitoring of the nutritional state with therapy is also imperative.

### Lifestyle Modifications in Patients With Severe EPI

Patients with severe pancreatic disease and marginal digestive capacity will require a number of lifestyle changes to help optimize the nonpancreatic organ functions. Key recommendations include

a. consuming frequent small meals without skipping any of them,
b. consuming high protein foods,
c. avoiding alcohol and tobacco, and
d. PERT supplementation.

For those with diabetes, particular attention to glycemic control is required and specialist dietetic support is indicated. Specifically, it will likely be necessary to minimize high sugar / high glycemic foods and drinks, to keep a diary of diet, glucose levels, PERT compliance and physical exercise, and schedule frequent dietitian assessments and monitoring, until stable. This is especially important in patients with glucose intolerance or diabetes mellitus (DM), as it is important to differentiate Type 2 DM (beta cell dysfunction / insulin resistance) from Type 3c DM (loss of islet cell mass).^120^^,^^121^ The benefits of a structured physical exercise program cannot be overstated in this setting and is data-supported. Studies confirm that resistance exercise in pancreatic cancer can enhance anabolism and maintain lean body mass and decrease sarcopenia.^122^^,^^123^ This outcome can easily be extrapolated to chronic pancreatitis. Although there have been few intervention studies investigating the benefits of exercise in chronic pancreatitis (constituting a critical research gap), data for other chronic diseases consistently show the benefits of physical activity.

## Conclusion: Advances and Gaps and a Draft Mechanistic Definition of EPI

This symposium provides an assessment of the current state of the art in the diagnosis and management of exocrine pancreatic insufficiency. This condition remains challenging even to define and serious limitations in diagnostic testing and therapeutic options lead to clinical confusion and frequently less than optimal patient management.

### Summary of the Framework for Understanding EPI

Humans appear to possess far more acinar cell mass and digestive enzyme secretion than that is necessary for health (ie, the so-called physiologic reserve). Earlier studies suggested that 90% of acinar cell mass can be lost without developing severe fat maldigestion but moderately severe chronic pancreatitis was not included in these studies and some patients with end-stage chronic pancreatitis still had near normal fat absorption, demonstrating high variability in the integrated pathobiology of individual patients including adaptive capacity of the gut. Indeed, in studies of PERT in patients with documented insufficiency, most of the dietary fat is still absorbed on the placebo therapy (usually > 70%). The concept of insufficiency therefore needs to include not just acinar cell mass and digestive output but also the nutritional needs, the diet, and specific nutritional consequences that occur in patients with altered gut health, structure, or function.

### Draft Mechanistic Exocrine Pancreatic Insufficiency Definition

#### Clinical Definition

A simple clinical definition of EPI may be useful in some context. Clinically, EPI is defined as inadequate delivery of pancreatic digestive enzymes to meals to meet nutritional needs and is reversed with appropriate treatment.

However, this does not address either the context of the clinical question or the threshold reduction of pancreatic exocrine secretion contributing to the signs and symptoms of maldigestion and subsequent malnutrition. Based on the critical review of older data and consideration of new information, a two-part mechanistic EPI definition is proposed.

#### Essence

EPI is a disorder caused by failure of the pancreas to deliver a minimum/threshold level of specific pancreatic digestive enzymes to the intestine in concert with ingested nutrients, followed by enzymatic digestion of a series of individual snacks and meals over time to meet nutritional and metabolic needs, given (a) the specific macronutritional and micronutritional needs; (b) nutrient intake; (c) exocrine pancreatic function; and (d) intestinal anatomy, function, diseases, and adaptative capacity.

#### Characteristics

EPI is characterized by variable deficiencies in micronutrients and macronutrients, especially essential fats and fat-soluble vitamins, by gastrointestinal symptoms of nutrient maldigestion and by improvement or correction of nutritional state with lifestyle changes, disease treatment, optimized diet, dietary supplements, and/or administration of adequate PERT.

The new mechanistic EPI definition addresses the major factors that affect the threshold for EPI, recognizes that this threshold differs for various micronutrients and macronutrients in different people, and that digestive system compensation or deficits that affect both the threshold for EPI and therapeutic strategies. The characteristics of EPI are important for both detection and treatment, including addressing other factors that cause EPI-like signs and symptoms.
