# Supplementary material for: AGA-PancreasFest Joint Symposium on Exocrine Pancreatic Insufficiency
Source: Gastro Hep Adv. 2022 Nov 15;2(3):395–411. doi: 10.1016/j.gastha.2022.11.008 (PMC11307793; doi:10.1016/j.gastha.2022.11.008)
Supplement: Supplemental information [file mmc1.docx]

**Supplemental Information.**

**Organization and Agenda of the *AGA-PancreasFest Joint Symposium on Exocrine Pancreatic Insufficiency***

The AGA-PancreasFest Joint Symposium represented the final session of PancreasFest 2021. The program was organized by members of the Program Committee (below) in concert with the Symposium co-directors, David C. Whitcomb MD PhD (representing PancreasFest) and Vivek Kaul, MD (representing the American Gastroenterology Association, AGA), and the Program Coordinator, Joy Merusi MA.

**PancreasFest 2-19 Program Committee:**

- Pittsburgh Regional Organizing Committee:
  - Kathryn Albers, PhD (Univ. of Pittsburgh Neuroscience)
  - Nathan Bahary, MD, PhD (Univ. of Pittsburgh Oncology) – *Course Director*
  - Randall E. Brand, MD (Univ. of Pittsburgh GI) – *Course Director*
  - Anna Evans Phillips, MD, MSc (Univ. of Pittsburgh GI)
  - Jami Saloman, PhD (Univ. of Pittsburgh GI)
  - Aatur Singhi, MD, PhD (Univ. of Pittsburgh Pathology)
  - David C. Whitcomb, MD, PhD (Univ. of Pittsburgh GI) – *Course Director*
  - Dhiraj Yadav, MD, MPH (Univ. of Pittsburgh GI)
- Maisam Abu-El-Haija, MD, MS (Cincinnati Children’s)
- Dana Andersen, MD (NIDDK/NIH) – (*ex officio* member)
- Michelle A. Anderson, MD, MSc (Univ. of Michigan GI)
- Darwin Conwell, MD, MS (Ohio State Univ. GI)
- Gregory Coté, MD, MSc (MUSC GI) – *Course Director*
- Zobeida Cruz-Monserrate, PhD (Ohio State Univ. GI)
- Guy E. Groblewski, PhD (Univ. of Wisconsin)
- Steven J. Hughes, MD (Univ. of Florida) – *Course Director*
- Mark E. Lowe, MD, PhD (Washington Univ. St. Louis)
- Stephen Pandol, MD (UCLA)
- Georgios Papachristou, MD, PhD (OSU)
- Walter Park, MD, MS (Stanford)
- Miklos Sahin-Toth, MD, PhD (UCLA)
- Aliye Uc, MD (Univ. of Iowa Pediatrics) – *Course Director*

Speakers represented established thought and opinion leaders from the United States and Ireland based on the spectrum of topics selected. The proposed agenda was reviewed and approved by the PancreasFest Organizing Committee and the AGA Institute (<https://gastro.org/meetings-and-learning/aga-endorsement/>). The program was also reviewed by the Center for Continuing Education in the Health Sciences of the University of Pittsburgh for CME credit. The University of Pittsburgh School of Medicine is accredited by the Accreditation Council for Continuing Medical Education (ACCME) to provide continuing medical education for physicians.

**Symposium Schedule**

12:15 **Introduction and Session Goals**

David C. Whitcomb, MD, PhD and Vivek Kaul, MD

12:25 **Session X: Definitions and Assessment of Exocrine Pancreatic Insufficiency (EPI)**

Moderator: Sohail Husain, MD

|  |  | 12:25 **Exocrine Pancreatic Insufficiency and Malnutrition** |
| --- | --- | --- |
|  |  | David C. Whitcomb, MD, PhD |
|  |  | 12:35 **Fat Digestion and Absorption** |
|  |  | Mark E. Lowe, MD, PhD |
|  |  | 12:50 **CF Nutritional Status: Impact of new CFTR and Nutrition Treatments** |
|  |  | Virginia A. Stallings, MD |
|  |  | 1:05 **Nutrition Management of Chronic Pancreatitis** |
|  |  | Sinead N. Duggan, RD, PhD |
|  |  | 1:20 **Discussion** |
| 1:30 |  | **Session XI: Extra-pancreatic Variables Affecting Digestion, Absorption, and Nutrition** |
|  |  | Moderator: Georgios I. Papachristou, MD, PhD |
|  |  |  |
|  |  | 1:30 **Intestinal Adaptation to Exocrine Pancreatic Insufficiency and Surgery** |
|  |  | Robert Martindale, MD, PhD |
|  |  | 2:00 **Celiac Disease, SIBO, and Other Disorders Confounding EPI** |
|  |  | Jodie A. Barkin, MD |
|  |  | 2:15 **Discussion** |
| 2:40 |  | **Session XII: Diagnosis and Treatment** |
|  |  | Moderator: Christopher Forsmark, MD |
|  |  | 2:40 **Pancreatic Function Testing** |
|  |  | Darwin Conwell, MD, MS |
|  |  | 3:00 **Treatment Strategies and Goals** |
|  |  | Vivek Kaul, MD |
|  |  | 3:20 **Discussion** |
| 3:30 |  | **Session XIII: Mechanistic Definition of EPI** |
|  |  | Moderators: David C. Whitcomb, MD, PhD and Vivek Kaul, MD |
|  |  | 3:30 **Description of a Mechanistic Definition of EPI** |
|  |  | David C. Whitcomb, MD, PhD |
|  |  | 3:45 **Discussion** |
|  |  | Panel and Audience |
| 4:30 |  | **Adjourn PancreasFest 2021** |
